# Bacterial Community Composition Associated with Pyrogenic Organic Matter (Biochar) Varies with Pyrolysis Temperature and Colonization Environment

**DOI:** 10.1128/mSphere.00085-17

**Published:** 2017-03-29

**Authors:** Zhongmin Dai, Albert Barberán, Yong Li, Philip C. Brookes, Jianming Xu

**Affiliations:** a Institute of Soil and Water Resources and Environmental Science, Zhejiang Provincial Key Laboratory of Subtropical Soil and Plant Nutrition, Zhejiang University, Hangzhou, China; b Department of Land, Air, and Water Resources, University of California, Davis, Davis, California, USA; University of Illinois at Urbana-Champaign

**Keywords:** *Actinobacteria*, bacterial community composition, *Chloroflexi*, pyrogenic organic matter, pyrolysis temperature, easily mineralizable carbon

## Abstract

Microbes that colonize pyrogenic organic matter (PyOM) (also called biochar) play an important role in PyOM mineralization and crucially affect soil biogeochemical cycling, while the microbial community composition associated with PyOM particles is poorly understood. We generated two manure-based PyOMs with different characteristics (PyOM pyrolyzed at the low temperature of 300°C [i.e., PyOM300] and at the high temperature of 700°C [i.e., PyOM700]) and added them to high-carbon (4.15%) and low-C (0.37%) soil for microbial colonization. 16S rRNA gene sequencing showed that *Actinobacteria*, particularly *Actinomycetales*, was the dominant taxon in PyOM, regardless of the PyOM pyrolysis temperature and soil type. Bacterial communities associated with PyOM particles from high-C soils were similar to those in non-PyOM-amended soils. PyOM300 had higher total microbial activity and more differential bacterial communities than PyOM700. More bacterial operational taxonomic units (OTUs) preferentially thrived on the low-pyrolysis-temperature PyOM, while some specific OTUs thrived on high-pyrolysis-temperature PyOM. In particular, *Chloroflexi* species tended to be more prevalent in high-pyrolysis-temperature PyOM in low-C soils. In conclusion, the differences in colonized bacterial community composition between the different PyOMs were strongly influenced by the pyrolysis temperatures of PyOM, i.e., under conditions of easily mineralizable C or fused aromatic C, and by other properties, e.g., pH, surface area, and nutrient content.

**IMPORTANCE** Pyrogenic organic matter (PyOM) is widely distributed in soil and fluvial ecosystems and plays an important role in biogeochemical cycling. Many studies have reported changes in soil microbial communities stimulated by PyOM, but very little is known about the microbial communities associated with PyOM. The microbes that colonize PyOMs can participate in the mineralization of PyOM, so changing its structure affects the fate of PyOMs and contributes to soil biogeochemical cycling. This study identified the bacterial community composition associated with PyOMs on the basis of high-throughput sequencing and demonstrated that both PyOM pyrolysis temperature and the colonization environment determined the bacterial community composition. Our work increases our understanding of the dominant phylogenetic taxa associated with PyOMs, demonstrates mechanisms mediating microbial metabolism and growth in PyOMs, and expands a new research area for pyrogenic organic matter. This study identified the bacterial community composition associated with PyOM, which is widely distributed in the environment. Most bacterial OTUs preferentially thrived on PyOM pyrolyzed at low temperature, while some specific OTUs thrived on PyOM pyrolyzed at high temperature.

## Abstract (precis)

Pyrogenic organic matter (PyOM) is widely distributed in soil and fluvial ecosystems and plays an important role in biogeochemical cycling. Many studies have reported changes in soil microbial communities stimulated by PyOM, but very little is known about the microbial communities associated with PyOM. The microbes that colonize PyOMs can participate in the mineralization of PyOM, so changing its structure affects the fate of PyOMs and contributes to soil biogeochemical cycling. This study identified the bacterial community composition associated with PyOMs on the basis of high-throughput sequencing and demonstrated that both PyOM pyrolysis temperature and the colonization environment determined the bacterial community composition. Our work increases our understanding of the dominant phylogenetic taxa associated with PyOMs, demonstrates mechanisms mediating microbial metabolism and growth in PyOMs, and expands a new research area for pyrogenic organic matter. This study identified the bacterial community composition associated with PyOM, which is widely distributed in the environment. Most bacterial OTUs preferentially thrived on PyOM pyrolyzed at low temperature, while some specific OTUs thrived on PyOM pyrolyzed at high temperature.

## INTRODUCTION

Pyrogenic organic matter (PyOM) produced by pyrolysis (usually called biochar) or natural fires is widely distributed in agricultural lands (1), wildfire-affected forests (2), and fluvial ecosystems (3). PyOM can exist in soil for thousands of years (4) and can constitute up to 80% of total soil organic carbon (5). In general, PyOM is intentionally applied to enhance soil carbon sequestration and improve soil fertility (6, 7). Recently, the research emphasis has been gradually switching from the application effects of PyOM (8–10) to the microbially mediated mechanisms of C and N cycling (11, 12). Some studies have already reported how PyOM alters soil microbial abundance and diversity and how changes in microbial community composition affect soil C and nutrient dynamics (13–15). For example, Whitman et al. (2016) (15) found that the relative abundance of *Gemmatimonadetes* tended to increase only in response to PyOM and not in response to fresh organic matter. Xu et al. (2014) (14) showed that the presence of PyOM increased bacterial diversity and the relative abundance of *Bacteroidetes*, *Gemmatimonadetes*, and TM7 and decreased the relative abundance of *Acidobacteria* and *Chloroflexi*.

PyOM has distinctive properties such as high biochemical stability, a highly porous structure, high alkalinity, and special nutrient composition (16, 17). In addition, PyOM is a heterogeneous material. PyOM properties differ greatly as pyrolysis conditions and feedstocks change (18). For instance, the C content, pH, and surface area of manure-based biochar increased from 29%, 8.5, and 4.8 m^2^·g^−1^ to 38%, 10.1, and 32.7 m^2^·g^−1^, respectively, when the pyrolysis temperature was increased from 300°C to 700°C (19). The distinctive properties of PyOM can favor specific microbial colonization and probably result in a great differentiation of the microbial community between different PyOMs as well as between PyOM and the adjacent soil. After colonization of PyOM, microbes can use it as their habitats, participate in PyOM mineralization, and, finally, affect PyOM fate and contribute to soil C and N cycling (20). A few studies have shown how PyOM particles harbor microbial communities that are different from those harbored by soils (12, 21, 22). For example, *Proteobacteria* and *Actinobacteria* were more abundant in PyOM particles than in adjacent control soils (12). However, due to the insufficient number of studies and the wide diversity of PyOM types, the different microbial community compositions associated with PyOMs are still poorly understood.

Two main factors might modulate microbial colonization in PyOMs: intrinsic properties (e.g., aromaticity, pH, surface area) and the surrounding environment. In general, the pyrolysis temperature greatly affects PyOM properties (23): low pyrolysis temperatures result in PyOM with more easily mineralizable C, lower pH, lower surface area, etc., while high pyrolysis temperatures result in PyOM with higher levels of fused aromatic C, higher pH, and greater surface area (24); such examples of PyOM are widely distributed in natural environments (25). Additionally, environmental conditions, e.g., soil physical and chemical characteristics (26), especially organic C status, can also influence PyOM-associated microbial communities (22).

In this study, we produced PyOMs at pyrolysis temperatures of 300°C and 700°C, using manure as feedstock, which is different from lignocellulose-based feedstock and yet is very commonly used in soil ecosystem studies. Two PyOMs were then added to two representative soils, a low-C-content soil (pH of 4.53, 0.37% C, and 0.06% N) (a Psammaquent [Ps] soil) and a high-C-content soil (pH of 5.55, 4.17% C, and 0.35% N) (an Argiustoll [Ar] soil) to investigate, by 16S rRNA gene sequencing, the community composition of bacteria colonized in PyOMs. Our objectives were (i) to gain comprehensive information concerning bacterial community composition associated with PyOM particles and to determine the differences between PyOM particles and non-PyOM-amended soils (also called control soils) and (ii) to elucidate how the PyOM pyrolysis temperature and the colonization environment affect bacterial community composition. We hypothesized that the presence of a low-pyrolysis-temperature PyOM would lead to larger differences in bacterial community composition than those seen with control soil, due to the availability of more easily mineralizable C together with other distinctive properties (e.g., pH, surface area, etc.) whereas high-pyrolysis-temperature PyOM would also harbor a specific microbial community due to its fused aromatic structure.

## RESULTS

### PyOM properties.

**FIG 1:**
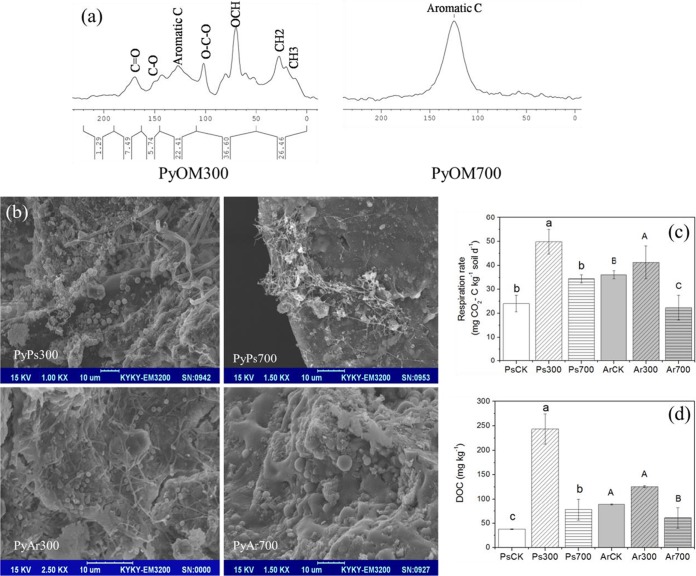
Relevant microbial biomass and activity parameters under PyOM300 and PyOM700 conditions. (a) NMR spectra. (b) SEM spectra. (c) Respiration rates. d, day. (d) DOC concentrations. Different lowercase and uppercase characters represent significant differences (*P* < 0.05) in the results seen with Ps and Ar treatments, respectively. SEM images were taken at the end of the incubation experiment to show the microbial colonization in PyOM particles that were extracted from soils.

The molar H/C (the higher the molar H/C, the lower the degree of fused aromatic C) in PyOM300 was higher than in PyOM700 (Table 1). PyOM300 had a higher proportion of volatile matter (48.0%) than PyOM700 (8.4%), whereas the fixed proportion of carbon in PyOM300 (15.3%) was lower than that in PyOM700 (35.7%) (Table 1). This result was consistent with nuclear magnetic resonance (NMR) spectra showing that the aromatic C was the dominant structure in PyOM700 whereas there were a variety of examples of aliphatic C, e.g., carbonyls (e.g., C=O and C-O) and alkyls (e.g., OCH, CH2, and CH3), detected in PyOM300 in addition to aromatic C (Fig. 1a). The pH and surface area of PyOM300 (7.2 and 4.9 m^2^·g^−1^) were lower than those of PyOM700 (9.6 and 52.6 m^2^·g^−1^), while the N mineral contents (i.e., NH_4_^+^ and NO_3_^−^) in the two PyOMs were similar (Table 1).

**TABLE 1:** Basic properties of PyOM300 and PyOM700

PyOM type	pH	Volatile matter (%)	Fixed carbon (%)	C (%)	H (%)	N (%)	Molar H/C	BET^a^ (m^2^ g^−1^)	Extractable NH_4_^+^ (mg kg^−1^)	Extractable NO_3_^−^ (mg kg^−1^)
PyOM300	7.2	48.0	15.3	35.3	4.48	3.03	1.52	4.9	40.2	18.5
PyOM700	9.6	8.4	35.7	29.9	1.40	1.64	0.56	52.6	16.1	8.6

a BET, Brunauer-Emmett-Teller surface area.

### Total microbial abundance and activity.

At the end of a 240-day incubation, numerous microbial cells were observed in PyOM300, whereas only a small number were observed in PyOM700, regardless of soil type (Fig. 1b and Fig. S1 in the supplemental material). Two-way analysis of variance (ANOVA) showed that the PyOM type significantly influenced soil microbial respiration and the dissolved organic carbon (DOC) concentration. PyOM300 addition significantly increased the soil microbial respiration rate in both Psammaquent (Ps) soil and Argiustoll (Ar) soil, and the increase in the Ps soil was larger than in the Ar soil. PyOM700 addition had no effects on the respiration rate in the Ps soil and significantly decreased the respiration rate in the Ar soil (Fig. 1c). Similarly, PyOM300 addition had larger effects on soil DOC concentrations than PyOM700 addition, and the increase in the Ps soil was larger than in the Ar soil (Fig. 1d).

10.1128/mSphere.00085-17.2 FIG S1  SEM spectra of the microbial colonization in PyOM300 and PyOM700. Download FIG S1, PDF file, 0.2 MB. Copyright © 2017 Dai et al. 2017 Dai et al. This content is distributed under the terms of the Creative Commons Attribution 4.0 International license.

### Bacterial composition.

**FIG 2:**
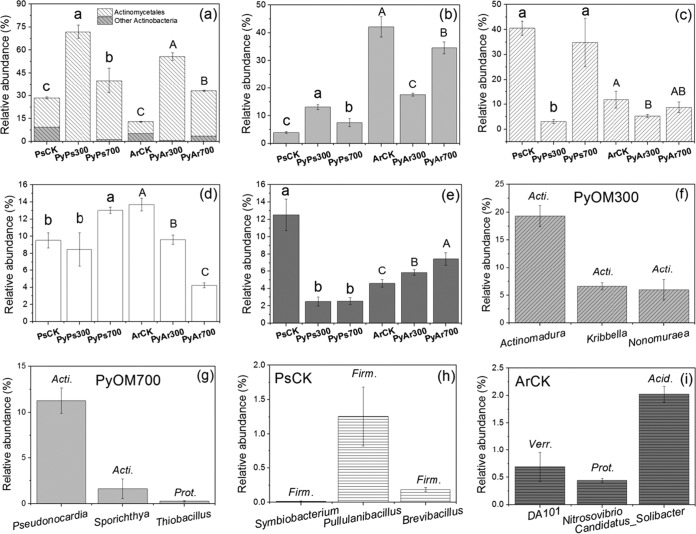
Relative abundances of dominant phyla in each treatment and indicator genera in PyOM300, PyOM700, and control soils. Values in Ps and Ar soil samples followed by the same lowercase and uppercase letter, respectively, are not significantly different at a *P* value of <0.05. (a) *Actinobacteria*. (b) *Proteobacteria*. (c) *Chloroflexi*. (d) *Acidobacteria*. (e) *Firmicutes*. (f) Indicator genera in PyOM300, regardless of soil type. (g) Indicator genera in PyOM700, regardless of soil type. (h) Indicator genera in PsCK. (i) Indicator genera in ArCK. *Acti.*, *Actinobacteria*; *Firm.*, *Firmicutes*; *Prot.*, *Proteobacteria*; *Acid.*, *Acidobacteria*; *Verri.*, *Verrucomicrobia*.

The dominant phylum in PyOMs was *Actinobacteria* (with an average relative abundance of 50.1%), followed by *Proteobacteria* (18.2%), *Chloroflexi* (13.0%), and *Acidobacteria* (8.8%), regardless of PyOM type and soil type (Fig. 2a to d). In addition, the relative abundances of *Actinobacteria* in PyOMs (71.8% for PyPs300 [PyOM300 extracted from Psammaquent soil], 40.0% for PyPs700 [PyOM700 extracted from Psammaquent soil], 55.7% for PyAr300 [PyOM300 extracted from Argiustoll soil], and 33.2% for PyAr700 [PyOM700 extracted from Argiustoll soil]) were significantly higher than those in the control soils (28.5% for Psammaquent soil without PyOM addition [PsCK] and 12.9% for Argiustoll soil without PyOM addition [ArCK]), and the abundance in PyOM300 (i.e., PyPs300 and PyAr300) was significantly higher than in PyOM700 (i.e., PyPs700 and PyAr700) (Fig. 2a). Consistently, at the order level, *Actinomycetales*, from the phylum of *Actinobacteria*, was the most dominant member in all PyOMs (with an average relative abundance of 48.7%) and was more dominant in PyOM300. Two-way ANOVA showed that PyOM type and soil type significantly affected the relative abundance of *Actinobacteria*. In addition, with respect to the PyOMs extracted from Ps soil, PyPs300 had significantly higher relative abundances of *Proteobacteria* and lower relative abundances of *Chloroflexi* and *Acidobacteria* than PyPs700 (Fig. 2b to d). With respect to the PyOMs extracted from Ar soil, PyAr300 had significantly higher relative abundances of *Acidobacteria* and lower relative abundances of *Proteobacteria* and *Firmicutes* than PyAr700 (Fig. 2b, d, and e). Indicator genera in PyOM300 and PyOM700 (irrespective of soil type) and in the control soils are shown in Fig. 2f to i. Most of the indicators in PyOM300 and PyOM700 were from the phylum *Actinobacteria*. The indicators with the highest abundances in PyOM300 and PyOM700 were *Actinomadura* (*Actinobacteria*) (Fig. 2f) and *Peseudonocardia* (*Actinobacteria*) (Fig. 2g), respectively. The indicator genera with the highest abundances of PsCK and ArCK were *Pullulanibacillus* (*Firmicutes*) (Fig. 2h) and “*Candidatus* Solibacter” (*Acidobacteria*) (Fig. 2i).

### Bacterial diversity and community similarity patterns.

**FIG 3:**
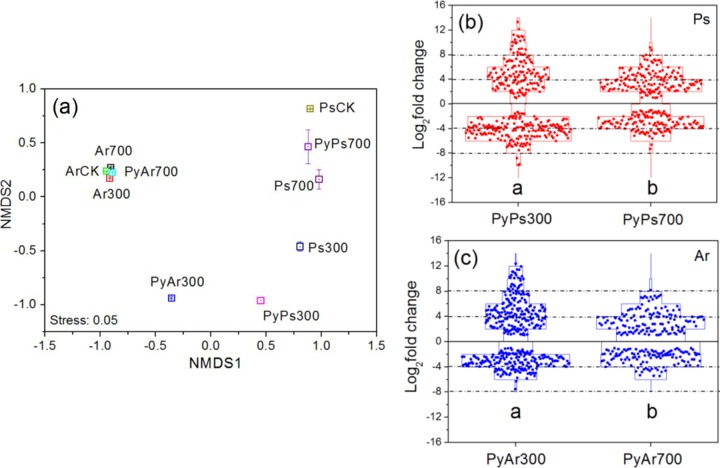
(a) Nonmetric multidimensional scaling plots (NMDS) of bacterial community patterns in the PyOMs, control soils, and PyOM-amended soils based on Bray-Curtis metric using OTU abundances. (b and c) Log_2_-fold change of differential abundant OTUs in PyOMs extracted from Ps soil and Ar soil, respectively. Ps300, Ps soil amended with PyOM300; Ps700, Ps soil amended with PyOM700; Ar300, Ar soil amended with PyOM300; Ar700, Ar soil amended with PyOM700. PyOM-amended soils in this figure can to some extent show the effect of the transition between PyOM particles and control soils. Other abbreviations are described in the main text. PERMANOVA showed the statistical significance of differences between treatments in community composition as follows: for PyPs300 versus PsCK, *P* < 0.05; for PyPs700 versus PsCK, *P* > 0.05; for PyAr300 versus ArCK, *P* < 0.05; for PyAr700 versus ArCK, *P* > 0.05; for PyPs300 versus PyPs700, *P* < 0.05; for PyAr300 versus PyAr700, *P* < 0.05. Different lower letters represent significant differences (*P* < 0.05) in the Log_2_-fold change of OTUs in PyOM300 and PyOM700 by *t* test.

Permutational multivariate analysis of variance (PERMANOVA) showed that both PyOM type (*R*^2^ = 0.63, *P* < 0.01) and soil type (*R*^2^ = 0.66, *P* < 0.01) significantly affected community similarity patterns (Fig. 3a). Bacterial taxonomic community composition in PyPs300 separated largely from that in the control soil (PsCK) (*P* < 0.05 [by PERMANOVA]), and the same trend was observed with PyAr300. However, PyPs700 and PyAr700 showed no significant differences in bacterial community composition from PsCK and ArCK, respectively (*P* > 0.05) (Fig. 3a). Also, PyPs300 and PyAr300 had significant differences in bacterial community composition from PyPs700 and PyAr700, respectively (*P* < 0.05) (Fig. 3a). All the Ar treatments clustered together (excluding the PyAr300 treatment), while the Ps treatments showed a more dispersed pattern (Fig. 3a).

**FIG 4:**
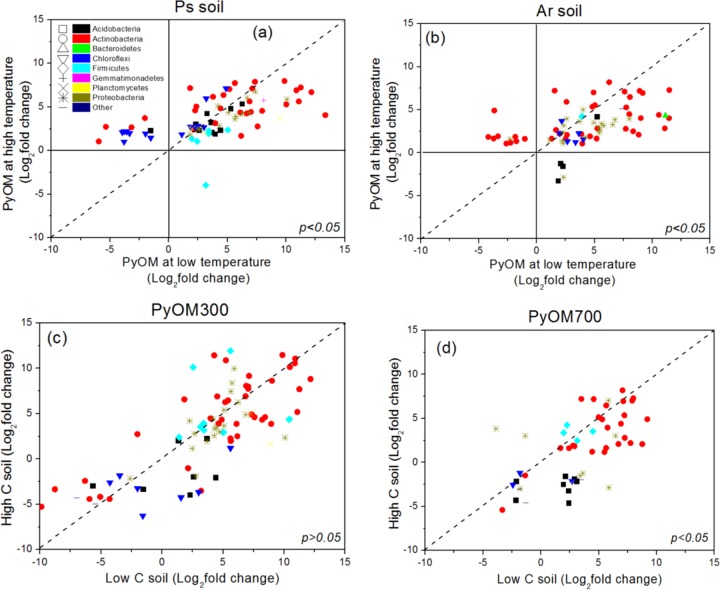
Log_2_-fold change in relative abundance of differentially abundant OTUs in PyOM300 and PyOM700 (a and b) and in Ps soil and Ar soil (c and d). Each dot represents a single OTU. Differentially abundant OTUs that were not significantly different (with Log_2_-fold change of <1 or adjusted *P* values of >0.1) compared with soil control are not presented in each figure. Dashed lines indicate that the preferences of OTUs for low- and high-pyrolysis-temperature PyOMs were equivalent. The OTUs indicated below the dashed lines preferred low-pyrolysis-temperature PyOM to high-pyrolysis-temperature PyOM, whereas the OTUs indicated above the dashed lines showed the opposite trend. *t* tests were conducted to show the statistically significant differences (*P* < 0.05) in the values of Log_2_-fold change between two PyOMs and between two soils. *P* values of <0.05 in panels a and b indicate that the whole bacterial community tended to thrive better on low-pyrolysis-temperature PyOM than on high-pyrolysis-temperature PyOM. *P* values of <0.05 in panels c and d indicate that the bacterial community tended to be more susceptible to being changed in low-C soil than in high-C soil.

As the two different soils had different original bacterial community compositions, we identified the bacterial OTUs in the PyOMs whose relative abundances were twice as large as in the control soils (Fig. 3b and c; see also Table S1 in the supplemental material). The proportions of total differentially abundant OTUs in PyOM300 (58.2% in PyPs300 and 57.0% in PyAr300) were larger than those in PyOM700 (48.7% in PyPs700 and 33.6% in PyAr700) (Table S1); therefore, PyOM300 had a greater number of differentially abundant OTUs than PyOM700. *t* test analysis showed that the Log_2_-fold change of OTUs in PyOM300 was significantly larger than that in PyOM700 (*P* < 0.05) (Fig. 3b and c) and therefore that the relative abundance of OTUs in PyOM300 differed more from that in the control soil than did that in PyOM700. Most of the OTUs preferred PyOM300 (Fig. 4a and b), while a small number of OTUs preferred PyOM700, especially the OTUs from phyla *Chloroflexi and Actinobacteria* in PyOMs from Ps soil (Fig. 4a) and from *Actinobacteria* in PyOMs from Ar soil (Fig. 4b).

10.1128/mSphere.00085-17.4 TABLE S1  The proportion of nondifferentially abundant OTUs and differentially abundant OTUs with each phylum in PyOM300 and PyOM700 incubated in two soils. Download TABLE S1, PDF file, 0.02 MB. Copyright © 2017 Dai et al. 2017 Dai et al. This content is distributed under the terms of the Creative Commons Attribution 4.0 International license.

## DISCUSSION

### Total microbial activity.

In general, PyOM is often modeled as two carbon pools: a persistent C pool and a labile C pool (27). Low pyrolysis temperature results in PyOM that contains more easily mineralizable C (28), which is readily utilized by microorganisms. High pyrolysis temperature induces a large fraction of fused aromatic C that is difficult for microorganisms to oxidize (29, 30). In this study, total microbial activity, as indicated by qualitative scanning electron microscopy (SEM) spectra and CO_2_ respiration rate and DOC concentration data, in PyOM300 was significantly higher than in PyOM700. This result is very consistent with previous studies where the respiration rates in PyOMs and/or PyOM C mineralization rates were higher with decreasing pyrolysis temperature (31–34). For instance, Luo et al. (2011) (34) used ^13^C isotope tracing and found that only 0.14% of PyOM at a pyrolysis temperature of 700°C was mineralized after 87 days of soil incubation, while a significantly higher proportion (i.e., 0.61%) of PyOM was mineralized at 350°C. Thus, the presence of easily mineralizable C, as a substrate for bacteria to consume and proliferate, is considered to be one of the principal factors determining the total microbial activity. Although other properties of PyOM (e.g., pH) that play important roles in microbial activity changes should not be neglected, easily mineralizable C is more important because most bacteria can use it as an energy source.

### Bacterial composition responds to PyOM pyrolysis temperature.

Although some studies have reported that the easily mineralizable C fraction has a great effect on total microbial activity, how the easily mineralizable C fraction and other PyOM characteristics affect microbial community composition remains unknown. Our results showed that the bacterial composition in PyOM300 is clearly different from that in PyOM700 and control soil. In addition, PyOM300 had a larger proportion of differentially abundant OTUs and larger changes in abundance of these OTUs than PyOM700 (Fig. 3b and c and 4a and b; see also Table S1 in the supplemental material). Because the aliphatic C fraction of PyOM increases with decreasing pyrolysis temperature, these results likely indicated that the aliphatic C fraction of PyOMs plays an important role in determining bacterial community composition, which agrees with previous studies (35–37). For example, Whitman et al. (2016) (15) showed that PyOM caused fewer changes in bacterial community composition than aliphatic C-sufficient stover substrate in soils. In addition, the majority of OTUs that preferred aliphatic C were from members of the phyla *Actinobacteria* and *Proteobacteria* (Fig. 4), most of which are normally regarded as decomposers or heterotrophs (38, 39), further supporting the idea of the importance of easily mineralizable C in PyOMs.

More importantly, we also found that some specific OTUs tended to thrive on high-pyrolysis-temperature PyOMs. These OTUs may metabolize aromatic C (e.g., polycyclic aromatic hydrocarbons, xylenols, cresols, etc.) as energy sources rather than easily mineralizable C for their proliferation and growth. To date, many studies have indicated that some specific microbes can utilize aromatic C as an energy source (40–43). For example, Griebler et al. (2004) (42) evaluated the intrinsic bioremediation potential in a tar oil-contaminated aquifer and showed some evidence for *in situ* biodegradation of aromatic hydrocarbons. We also found that *Chloroflexi* members preferentially thrived on high-pyrolysis-temperature PyOM (Fig. 4a) rather than low-pyrolysis-temperature PyOM. Generally, some *Chloroflexi* are known as anaerobic halorespiring bacteria that use halogenated organics as energy sources during redox reactions (44). Therefore, the graphite-like structure of high-pyrolysis-temperature PyOM can probably act as an electron transport shuttle (45) and the porous structure would provide more anaerobic sites for use by them to metabolize halogenated organics. Other OTUs which prefer high-pyrolysis-temperature PyOM are mainly from *Actinobacteria* and are discussed below.

Given that C sources, i.e., aliphatic C in low-pyrolysis-temperature PyOM or aromatic C in high-pyrolysis-temperature PyOM, can favor some specific OTUs over others, we assumed that the differences in the physicochemical properties of the two PyOMs would also attract some specific bacteria and benefit their microbial growth and proliferation. Some (but not all) of the potential mechanisms in addition to carbon sources are as follows. (i) High-pyrolysis-temperature PyOM had a more porous structure and a greater surface area than low-pyrolysis-temperature PyOM (Table 1), features which can directly attract some specific microbes, e.g., filamentous bacteria, to colonize or can protect them from predators in surrounding soils (6). In addition, the porous structure can also absorb soluble organic matter, gases, and mineral nutrients, which could provide a habitat for microbes to metabolize and grow. However, how pore size (i.e., that of macropores, mesopores, and micropores) selectively affects microbial colonization needs further investigation. (ii) pH is one of the most important environmental factors affecting bacterial relative abundance and diversity (46), and the peak diversity appeared in soils with near-neutral pHs (47). Given that the PyOM300 had a neutral (i.e., 7.2) pH (Table 1), we assumed that the greater differences in the abundances of OTUs in low-pyrolysis-temperature PyOM may be also attributable to the appropriate pH environment for microbial growth. (iii) The graphite-like structure of PyOM can promote electron transportation when anaerobic bacteria participate in redox reaction (45), and Yu et al. (2015) (48) demonstrated that the promotion ability of PyOM increased as the pyrolysis temperature increased. This is another possible reason why some specific OTUs preferentially thrived on PyOM700. (iv) High-pyrolysis-temperature PyOM has higher mineral content than low-pyrolysis-temperature PyOM, a feature which is essential for microbial growth and metabolism (10). As manure-based PyOM had high total N content compared to other PyOMs, there is an assumption that N content also affects microbial community composition. However, the N mineral content (NH_4_^+^ and NO_3_^−^) in PyOM300 was close to that in PyOM700 and comprised only a very small proportion of total N (Table 1). Thus, we do not attribute the differential preference of microbial colonization to the mineral N contents. However, the effects of other minerals (e.g., P, K, etc.) on microbial colonization need further investigation. Also, the potential mechanisms are likely to interact, and isolating all these different mechanisms is a great challenge that future studies will have to address.

### Bacterial composition responds to soil type.

As different colonization environments have different characteristics, e.g., different levels of original C content, we assumed that the bacterial community composition in high-C soil, which was more closely related to PyOMs, would not be much changed. In our study, the Ps soil, which has undergone high weathering (49), had a very low nutrient status, e.g., 0.37% organic C content (Table 2). In contrast, the Ar soil had undergone moderate weathering and had a high nutrient status, e.g., 4.15% organic C content (Table 2), which is closer to that in the two PyOMs (about 30%) than to that in the Ps soil (0.37%) (Table 1). Therefore, the Ar soil might provide sufficient carbon for microbial growth and metabolism. Thus, the bacterial community composition in the PyOMs added to the high-C soil did not greatly change, while the PyOMs from low-C soil had a much larger community composition change. Here, we should point out that the OTUs in low-pyrolysis-temperature PyOM (PyOM300) from high-C soil showed no significant difference from that from the low-C soils (*P* > 0.05) (Fig. 4c), suggesting that the easily mineralizable C fraction in PyOM300 probably had the overwhelming effects on microbial community composition change. This effect induced a great change in bacterial community composition even when the colonization environment was C sufficient. In contrast, the C status of high-pyrolysis-temperature PyOM (PyOM700) differed greatly from that in the low-C soil but was closer to that in the high-C soil, resulting in a larger change in the bacterial community in low-C soil than in high-C soil (Fig. 4d).

**TABLE 2:** Basic properties of Ps soil and Ar soil and PyOM extraction efficiency

Soil	pH	EBC (cmol kg^−1^)^a^	Total organic C (g kg^−1^)	Texture (clay/silt/sand) (%/%/%)	Total N (g kg^−1^)	% PyC300 extraction efficiency	% PyC700 extraction efficiency
Psammaquent	4.53	1.23	3.7	(7/24/69)	0.6	31	44
Argiustoll	5.55	10.00	41.5	(26/47/27)	3.5	25	40

a The total amounts of exchangeable base cations (EBC) were calculated as the sum of exchangeable K^+^, Na^+^, Ca2^+^, and Mg2^+^.

Our study for the first time demonstrated the potential mechanisms of the differential colonization of the bacterial community in PyOMs with different properties and incubated under different conditions, with results that were very different from those of previous studies that identified only the dominant phyla associated with one type of PyOM (12, 21, 22). Our results provide an advanced insight into the ecological contribution of each phylogenetic taxonomy at the OTU level to PyOM mineralization and soil biogeochemical cycling.

### *Actinobacteria* in PyOMs.

The phylum *Actinobacteria*, particularly the order *Actinomycetales*, comprising a group of Gram-positive aerobic bacteria with high G+C content in their DNA and primarily filamentous bacteria (50), had the highest relative abundance (with an average value of 50.1%) in the PyOMs, regardless of PyOM pyrolysis temperature and colonization environment. This phenomenon is consistent with a study by Taketani et al. (2013) (21). They reported that the abundance of *Actinobacteria* was higher in fire-burned wood PyOM from Amazonian dark earth than in dark earth itself. Sun et al. (2016) (12) also showed that the relative abundance of *Actinobacteria* in PyOM pellets was higher than that in adjacent control soils.

In general, *Actinobacteria* (especially *Actinomycetales*) are heterotrophic bacteria which play a major role in the decomposition of organic matter (39). The growth of *Actinobacteria* in PyOMs could be attributed to the fraction of easily mineralizable C in PyOMs as they were more abundant in low-pyrolysis-temperature PyOMs than in high-pyrolysis-temperature PyOM, which had more fused aromatic C. Whitman et al. (2016) (15) showed that *Actinomycetales* responded more strongly to stover addition (easily mineralizable C) than to PyOM, which contained only a small fraction of easily mineralizable C. Watzinger et al. (2014) (43) showed that PyOM was being mineralized by *Actinomycetales* in planosol soil during a short-term incubation. These studies to some extent showed that the C substrate is the principal factor mediating *Actinobacteria* growth in PyOMs. At the OTU level, although a large number of actinobacterial OTUs preferred aliphatic C, some OTUs from *Actinobacteria* (e.g., *Actinospicaceae*, *Pseudonocardia*, *Mycobacterium*) tended to thrive on PyOM700, the PyOM with high aromaticity (Fig. 4a and b). One possible reason is that some OTUs presumably preferentially used aromatic C as an energy source rather than easily mineralizable C. Moreover, due to the unique properties of PyOMs produced at high pyrolysis temperature, i.e., those with a highly porous structure and an extensive surface area, these PyOMs, providing an appropriate habitat, would stimulate *Actinobacteria* (especially *Actinomycetales*) growth on the basis of a sufficient carbon source supply. Although it is difficult to identify whether the hyphae and spores (Fig. S2) were from fungi or *Actinobacteria*, we can still predict that the porous structure and high surface area are likely to absorb microbial spores and to be particularly suitable for the colonization and growth of microbial hyphae (51).

10.1128/mSphere.00085-17.3 FIG S2  Possibility of the existence of spores (a) and hyphae (b) of *Actinobacteria* or fungi in the PyOMs revealed by SEM spectra. Download FIG S2, PDF file, 0.2 MB. Copyright © 2017 Dai et al. 2017 Dai et al. This content is distributed under the terms of the Creative Commons Attribution 4.0 International license.

Our study identified the differentially abundant taxa colonized in PyOMs compared with control soil at fine taxonomic resolution and investigated the potential ecological roles of the dominant taxa that would increase PyOM degradation and contribute to soil biogeochemical cycling. Due to PyOM heterogeneity, we also examined the possibility of the effects of different PyOM variances on bacterial community composition. We found that the changes in total microbial activity and bacterial community composition are a result of microbial differential preferences for carbon source of PyOM and other PyOM characteristics (e.g., pH, porosity of structure, surface area, and nutrients). More importantly, some specific OTUs tended to thrive on high-pyrolysis-temperature (e.g., 700°C) PyOM, which was likely attributable to the capacity of these OTUs to use aromatic C as well as to colonize the unique PyOM structure. Further studies should target the ecological role of each specific taxon associated with PyOMs and its role in PyOM mineralization and soil biogeochemical cycling.

## MATERIALS AND METHODS

### PyOM preparation and soil collection.

Dry autoclaved swine manure was pyrolyzed at temperatures of 300°C and 700°C and was designated PyOM300 and PyOM700, respectively. The feedstock was placed in stainless steel trays, covered with a tight lid, and pyrolyzed under oxygen-limited conditions in a muffle furnace. The heating rate was 2.5°C·min^−1^, and the residence time was 0.5 h. After pyrolysis, the PyOM samples were ground and sieved using pore sizes between <3 mm and >2 mm. (Preliminary experiments showed the PyOM particles with this size range were easily extracted from soils). Quantitative ^13^C solid-state nuclear magnetic resonance (NMR) spectroscopy (BrukeBiospin, Germany) was performed at a magic-angle spinning (MAS) frequency of 14 kHz to quantify the aliphatic and aromatic structures in PyOM300 and PyOM700. Other methods for analysis of PyOM characteristics are described in Text 1 in the supplemental material. The basic properties of the PyOMs are given in Table 1. Two types of soil, a Psammaquent soil (termed “Ps soil”) and an Argiustoll soil (termed “Ar soil”), were collected from upland cropland in the Jinqu Basin of Zhejiang Province, China, and from maize farmland in Heilongjiang Province, China, respectively. Soil samples were collected from the topsoil (depth, 0 to 10 cm) after surface organic residues were removed and were then air-dried, crushed, and sieved using pore sizes of <2 mm. The basic properties of the soils are given in Table 2.

10.1128/mSphere.00085-17.1 TEXT S1  Supplemental methods. Download TEXT S1, PDF file, 0.2 MB. Copyright © 2017 Dai et al. 2017 Dai et al. This content is distributed under the terms of the Creative Commons Attribution 4.0 International license.

### Microbial colonization.

To stimulate microbial activity, soils were preincubated aerobically (i.e., under open-air conditions) for 2 weeks at 25°C and at 30% water-holding capacity. Then, PyOM300 and PyOM700 were added to the soils at 30 g·kg^−1^ (3% [wt/wt]). The PyOM-soil mixtures (500 g soil and 15 g PyOM) were then incubated aerobically in plastic bags (the bags were slightly open in order to maintain adequate oxygen throughout) with three replicates, at 25°C in darkness at 70% water-holding capacity. We also set up the control soil without PyOM addition. The moisture of each treatment was readjusted by adding sterile water every 5 days. All the incubation processes were conducted in a superclean environment. After 240 days of incubation, the soils were homogenized and then subsampled for measurement of respiration rates and dissolved organic carbon (DOC) levels. Details of the methods used are provided in Text 1.

### PyOM extraction and SEM observation.

The method of PyOM extraction from soils was modified from that described previously by Lin et al. (2012) (52). Briefly, 200 g of soil PyOM mixtures was added to beakers, each with 1,000 ml of sterile water. The mixtures were then stirred gently for 2 min, and the isolated PyOMs in the suspension were collected on a sieve during the agitation. In this process, the PyOM pieces remain on the sieve surface and the soil particles pass through it. The PyOM particles were then collected manually, gently rinsed with sterile water to remove the residual soil particles, and then stored at −80°C (soil particles not able to be removed are considered the PyOM-sphere). The same washing treatments were also conducted in control soils. This extraction method is based on the assumption that the bacteria in the PyOMs were not damaged by water washing, which is similar to natural processes, e.g., rain leaching and water irrigation. The extraction rates are shown in Table 2. Scanning electron microscopy (SEM) (FEI, Netherlands) analysis was conducted to observe the colonization of microbes in the PyOMs that were extracted from soils using the method described by Dai et al. (2013) (17). All SEM figures presented in the manuscript were representatively selected, and other images were presented in Fig. S1 in the supplemental material.

### DNA extraction and sequencing and data processing.

We extracted DNA from extracted PyOMs and control soils using a Mo Bio PowerSoil DNA isolation kit (Mo Bio Laboratories, USA) following the manufacturer’s instructions. The amplification of the V3-V4 region of the 16S rRNA gene in each sample in triplicate was conducted using a ABI GeneAmp 9700 system (Thermo Fisher Scientific, Waltham, MA, USA) and TransStart Fastpfu DNA polymerase. The forward and reverse primers were 338F (5′-ACTCCTACGGGAGGCAGCA-3′) and 806R (5′-GGACTACHVGGGTWTCTAAT-3′), respectively. The detailed procedures were conducted by Shanghai Majorbio Bio-pharm Technology Co., Ltd., China. Triplicate amplified samples were then purified, pooled, and sequenced with a MiSeq PE300*2 sequencing platform (Illumina, USA). Overall, treatments with three replicates are abbreviated as follows: PsCK (Psammaquent soil without PyOM addition), PyPs300 (PyOM300 extracted from Psammaquent soil), PyPs700 (PyOM700 extracted from Psammaquent soil), ArCK (Argiustoll soil without PyOM addition), PyAr300 (PyOM300 extracted from Argiustoll soil), and PyAr700 (PyOM700 extracted from Argiustoll soil).

The raw data that contained two paired-end reads were processed with QIIME (version 1.7.0; http://qiime.org/) (53). Briefly, paired-end reads were combined to form tags based on overlaps and were then filtered, trimmed, and optimized as follows: (i) removal of bases with an average quality level of <Q20; (ii) minimal overlapping length of 10 bp; (iii) mismatching ratio of overlapped region of ≤0.2. Operational taxonomic units (OTUs) at a 97% similarity level were defined and clustered using USEARCH (version 7.1), and the taxonomy was assigned based on the Greengenes database (release 13.5; http://greengenes.secondgenome.com/) (54). All the sequencing data were resampled to the minimum sequencing depth of 30,000 reads. Raw data have been deposited in the GenBank Sequence Read Archive.

### Statistical analysis.

Two-way analysis of variance (ANOVA) was used to test for differential results caused by differences in pyrolysis temperature and soil type among the following treatments: (i) CO_2_ respiration rates and DOC concentrations and (ii) relative abundances of the dominant bacterial phyla. We explored bacterial community patterns using nonmetric mutidimensional scaling plots (NMDS) of taxonomic similarity (Bray-Curtis) and the vegan R package (55). Nonparametric PERMANOVA (permutational multivariate analysis of variance) was conducted to investigate the effects of soil and PyOM type on bacterial community similarity (56). The R package “DESeq2” (57) was used to identify those OTUs of which the relative abundance differed significantly from control soil results (58). In the data processing, the PsCK was designated the control for treatments of both PyPs300 and PyPs700, and the ArCK was designated the control for treatments of both PyAr300 and PyAr700. The selection criterion was based on *P* values adjusted by the Benjamini and Hochberg correction method. Thus, OTUs with a Log_2_-fold change in relative abundance of >1 and an adjusted *P* value of <0.1 were selected for further analysis and termed “differentially abundant OTUs.” These differentially abundant OTUs can provide in-depth information related to how the bacterial composition in PyOMs differed from that in control soil at a fine taxonomic resolution. *t* tests were conducted to show the significance of the difference (*P* < 0.05) in the values of Log_2_-fold change between two PyOMs and between two soils. Indicator genera representing the specialists that can be found in one type of PyOM but not in other PyOMs were identified using the indicator value metric (Indval) (59) as implemented in the labdsv R package (60).

### Accession number(s).

Raw sequencing read data have been deposited in the GenBank Sequence Read Archive under accession no. SRX1853875.
