# Supplementary figures and images for: Bacterial Community Composition Associated with Pyrogenic Organic Matter (Biochar) Varies with Pyrolysis Temperature and Colonization Environment
Source: mSphere. 2017 Mar 29;2(2):e00085-17. doi: 10.1128/mSphere.00085-17 (PMC5371693; doi:10.1128/mSphere.00085-17)

Fig. S1. SEM spectra of the microbial colonization in PyOM300 and PyOM700.

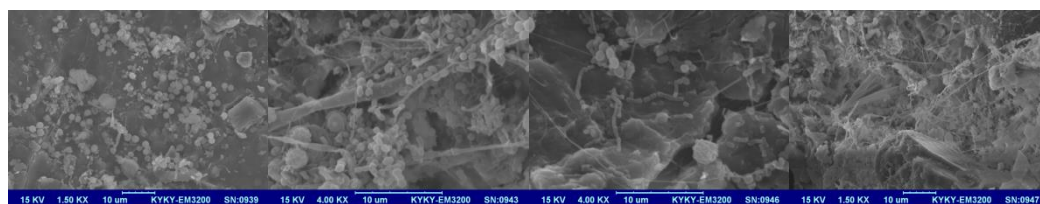

PyPs300

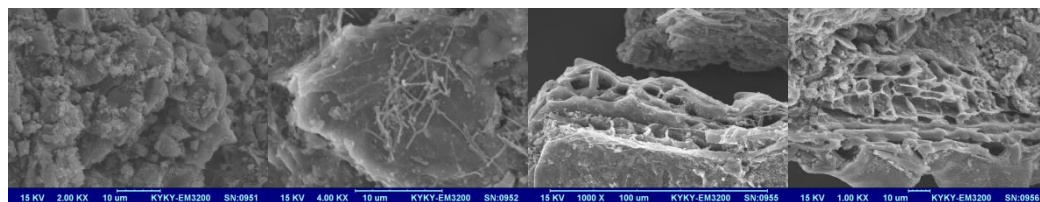

PyPs700

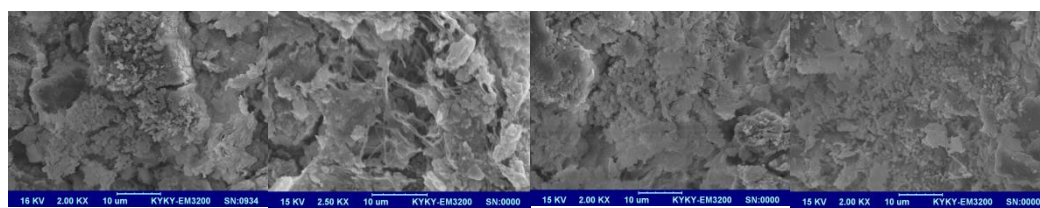

PyAr300

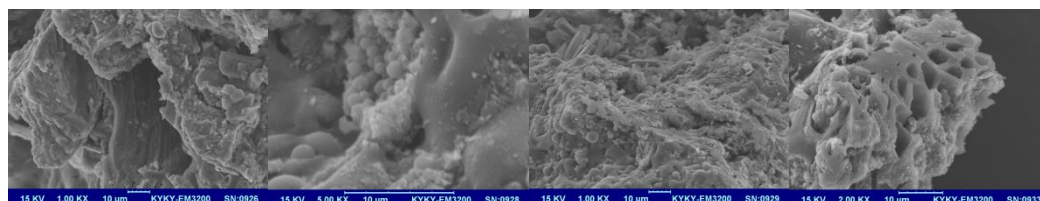

PyAr700

Supplement: FIG S1 [file sph002172259sf3.pdf]
