# Supplementary material for: Bacterial Community Composition Associated with Pyrogenic Organic Matter (Biochar) Varies with Pyrolysis Temperature and Colonization Environment
Source: mSphere. 2017 Mar 29;2(2):e00085-17. doi: 10.1128/mSphere.00085-17 (PMC5371693; doi:10.1128/mSphere.00085-17)
Supplement: TABLE S1 [file sph002172259st2.pdf]

Table S1. The proportion of non-differential abundant OTUs and differential abundant OTUs with each phylum in PyOM300 and PyOM700 incubated in two soils.

Proportion		PyPs300	PyPs700	PyAr300	PyAr700
Total non-differential abundant OTUs		41.8%	51.3%	43.0%	66.4%
Total differential abundant OTUs		58.2%	48.7%	57.0%	33.6%
Differential abundant OTUs in	*Acidobacteria*	4.6%	3.9%	4.8%	2.7%
	Actinobacteria	15.2%	11.2%	16.0%	10.2%
	Bacteroidetes	0.2%	0.2%	2.3%	1.6%
	Chloroflexi	20.4%	16.3%	8.1%	4.0%
	Firmicutes	6.2%	6.2%	1.8%	0.8%
	Gemmatimonadetes	0.7%	0.3%	4.1%	1.2%
	Planctomycetes	1.4%	0.8%	1.1%	0.5%
	Proteobacteria	7.8%	7.6%	15.7%	9.5%
	Others	1.6%	2.2%	3.1%	3.0%
