# Supplementary material for: Bacterial Community Composition Associated with Pyrogenic Organic Matter (Biochar) Varies with Pyrolysis Temperature and Colonization Environment
Source: mSphere. 2017 Mar 29;2(2):e00085-17. doi: 10.1128/mSphere.00085-17 (PMC5371693; doi:10.1128/mSphere.00085-17)

Fig. S2. Possibility of the existence of spores (a) and hyphae (b) of *Actinobacteria* or fungi in the PyOMs revealed by SEM spectra.

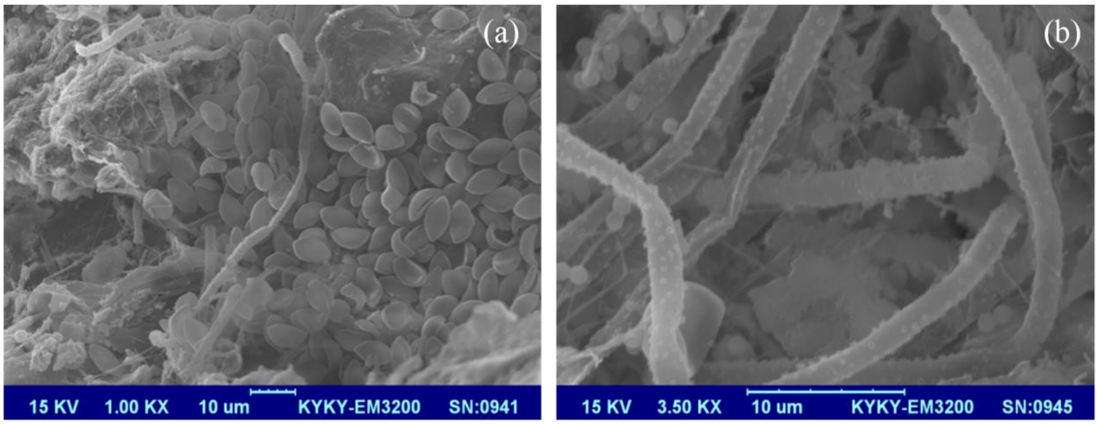

Supplement: FIG S2 [file sph002172259sf4.pdf]
