# Supplementary material for: Bacterial Community Composition Associated with Pyrogenic Organic Matter (Biochar) Varies with Pyrolysis Temperature and Colonization Environment
Source: mSphere. 2017 Mar 29;2(2):e00085-17. doi: 10.1128/mSphere.00085-17 (PMC5371693; doi:10.1128/mSphere.00085-17)
Supplement: TEXT S1 [file sph002172259s1.pdf]

Methods:

PyOM characteristics and soil properties

Proximate analysis (fixed carbon and volatile matter) of PyOM300 and PyOM700 were conducted using a modified method from ASTM D1762-84 Chemical Analysis of Wood Charcoal. PyOM pH was determined in deionized water at the ratio of 1:10 w/w PyOM/water. Surface area (BET) was measured by N_2 gas sorption analysis using a Nova 2200e surface area analyzer (Tristar3200, Micromeritics, USA) after degassing at 200 ℃ for a minimum of 8 h. Total C, H, and N concentrations of PyOMs were measured with a Flash EA 1112 elemental analyzer (Thermo Scientific, USA). Soil total organic C and soil dissolved organic C (DOC) were determined using a TOC/TN Analyzer (Analytik Jena AG, Jena, Germany). Soil total N concentration was also measured with a Flash EA 1112 elemental analyzer (Thermo Scientific, USA). At the end of incubation, we took the soil samples from each treatment and reincubated them for 7 days to measure the microbial respiration rate. Soil respiration rate was determined by titration with 0.25 M HCl after trapping of CO_2 in 10 ml aliquots of NaOH (0.5molL^{-1}) using phenolphthalein as titration indicator (Wang et al. 2013). Blank controls without soil were set up. The respiration rate was calculated from the average value of the measurement at 0 and 7 days. Exchangeable K^+, Na^+, Ca^{2+}, Mg^{2+} were extracted with 1 M ammonium acetate adjusted to pH 7.0. The K^+, Na^+, Ca^{2+} and Mg^{2+} were then measured by flame atomic absorption spectrometry (Analytikjena, Germany). The total exchangeable base cations (EBC) were calculated as the sum of exchangeable K^+, Na^+, Ca^{2+} and Mg^{2+}. Soil texture, i.e. sand, silt and clay content, was determined using the methods of Lu (2000). The extractable NH_4^+ and NO_3^- in the PyOMs were extracted with deionized water using a 1:10 PyOM/water ratio and then determine by the continuous flow analytical system (Skalar SAN++ System, Netherlands).

Reference:

Wang Y, Tang C, Wu J, Liu X, Xu J. 2013. Impact of organic matter addition on pH change of paddy soils. J Soils Sediments 13:12–23.

Lu RK. 2000. Soil Agro-Chemistry Analysis. Agricultural Technical Press of China, Beijing. p. 272–282.
